# Biomechanical Effects of Prefabricated Foot Orthoses and Rocker‐Sole Footwear in Individuals With First Metatarsophalangeal Joint Osteoarthritis

**DOI:** 10.1002/acr.22743

**Published:** 2016-04-27

**Authors:** Hylton B. Menz, Maria Auhl, Jade M. Tan, Pazit Levinger, Edward Roddy, Shannon E. Munteanu

**Affiliations:** ^1^ La Trobe UniversityBundooraVictoriaAustralia; ^2^ La Trobe University, Bundoora, Victoria, Australia, and Victoria UniversityMelbourneVictoriaAustralia; ^3^ Keele UniversityStaffordshireUK

## Abstract

**Objective:**

To evaluate the effects of prefabricated foot orthoses and rocker‐sole footwear on spatiotemporal parameters, hip and knee kinematics, and plantar pressures in people with first metatarsophalangeal (MTP) joint osteoarthritis (OA). *Methods*. A total of 102 people with first MTP joint OA were randomly allocated to receive prefabricated foot orthoses or rocker‐sole footwear. The immediate biomechanical effects of the interventions (compared to usual footwear) were examined using a wearable sensor motion analysis system and an in‐shoe plantar pressure measurement system.

**Results:**

Spatiotemporal/kinematic and plantar pressure data were available from 88 and 87 participants, respectively. The orthoses had minimal effect on spatiotemporal or kinematic parameters, while the rocker‐sole footwear resulted in reduced cadence, percentage of the gait cycle spent in stance phase, and sagittal plane hip range of motion. The orthoses increased peak pressure under the midfoot and lesser toes. Both interventions significantly reduced peak pressure under the first MTP joint, and the rocker‐sole shoes also reduced peak pressure under the second through fifth MTP joints and heel. When the effects of the orthoses and rocker‐sole shoes were directly compared, there was no difference in peak pressure under the hallux, first MTP joint, or heel; however, the rocker‐sole shoes exhibited lower peak pressure under the lesser toes, second through fifth MTP joints, and midfoot.

**Conclusion:**

Prefabricated foot orthoses and rocker‐sole footwear are effective at reducing peak pressure under the first MTP joint in people with first MTP joint OA, but achieve this through different mechanisms. Further research is required to determine whether these biomechanical changes result in improvements in symptoms.

## INTRODUCTION

Osteoarthritis (OA) is the most common musculoskeletal disorder in the world, affecting 10% of men and 13% of women age >60 years 1. Although the knee is the most commonly affected lower extremity region, foot involvement is also common. The most commonly affected region of the foot is the first metatarsophalangeal (MTP) joint, with radiographic changes evident in up to 35% of people age >35 years 2. The population prevalence of symptomatic radiographic first MTP joint OA (i.e., both radiographic changes and symptoms) in people age >50 years has recently been estimated as 7.8% 3. First MTP joint OA has a detrimental impact on health‐related quality of life 4, and 72% of those affected report associated locomotor disability 3.

Box 1 **Significance & Innovations**

- This is the first study to compare the biomechanical effects of foot orthoses and rocker‐sole shoes in people with first metatarsophalangeal (MTP) joint osteoarthritis.
- Both interventions were similarly effective at reducing pressure under the first MTP joint during gait, but achieved this through different mechanisms.
- Foot orthoses increased pressure under the midfoot and lesser toes.
- Rocker‐sole shoes decreased pressure under the second through the fifth MTP joints.

Structural and biomechanical factors are thought to contribute to the onset, progression, and symptomatic severity of first MTP joint OA. During the propulsive phase of gait, the first MTP joint dorsiflexes to assist in the forward transfer of bodyweight. However, in the presence of an overly long and/or wide first metatarsal or proximal phalanx, the proximal phalanx is unable to dorsally rotate on the first metatarsal head, resulting in joint compression and the development of a dorsal exostosis 5. In clinical practice, first MTP joint OA is often managed with foot orthoses, which are thought to decrease pain associated with this condition by allowing the first metatarsal to achieve sufficient plantarflexion in preparation for propulsion, thereby minimizing joint compression 6. Alternatively, pain relief can be achieved using a footwear modification known as a rocker‐sole, in which the sole of the shoe is curved 7. The aim of this modification is to allow the body's center of mass to “roll over” the base of support, reducing the need for first MTP joint dorsiflexion and subsequently decreasing the loads placed on the forefoot and toes.

Evidence pertaining to the proposed mechanism of action of foot orthoses in the treatment of first MTP joint OA is limited to a case series study of 9 participants who reported no change in first MTP joint dorsiflexion when the orthoses were worn 8. Studies of asymptomatic participants have been inconsistent, with 2 studies reporting a decrease in first MTP joint dorsiflexion (with medial wedging [9] and orthoses [10]) and a recent study demonstrating a small increase in the declination angle of the first metatarsal when participants wore an orthosis with material removed from beneath the first MTP joint 11. No studies have been undertaken to assess the biomechanical effects of rocker‐sole shoes in participants with first MTP joint OA, although studies in asymptomatic participants indicate a reduction in sagittal plane motion of the forefoot 12 and ankle 12, 13, 14, 15, reduced forefoot plantar pressures 15, 16, and reduced first MTP joint dorsiflexion 17 when walking compared to usual footwear.

Given the uncertainty regarding the mechanism of action of these 2 treatments, the objective of this study was to evaluate the immediate biomechanical effects of individualized, prefabricated foot orthoses and rocker‐sole shoes in individuals with first MTP joint OA. To do this, we conducted baseline kinematic and in‐shoe plantar pressure analyses of participants enrolled in a randomized trial 18 when wearing their usual footwear and their allocated intervention (i.e., orthoses or rocker‐sole shoes).

## MATERIALS AND METHODS

The data presented in this paper were collected at the baseline assessment of a larger randomized trial (Australian New Zealand Clinical Trials Registry ID: 12613001245785). The La Trobe University Human Ethics Committee provided ethical approval (13‐003), and all participants provided written informed consent prior to enrollment. The full trial protocol has been published previously 18.

#### Design

The study design was a parallel‐group, randomized trial comparing 2 interventions: prefabricated foot orthoses (Vasyli Custom, Vasyli Medical) versus commercially available rocker‐sole footwear (Masai Barefoot Technology [MBT], Matwa model). Permuted block randomization with random block sizes, stratified by sex, was undertaken using an interactive voice response telephone service provided by the National Health and Medical Research Council Clinical Trials Centre at the University of Sydney, New South Wales, Australia to ensure allocation concealment. Participants were informed that they would receive either the foot orthoses or rocker‐sole footwear (i.e., they were not blinded to their group allocation).

#### Participant recruitment, screening, and eligibility criteria.

To be included in the study, participants had to 1) be age ≥18 years; 2) report having pain in the first MTP joint on most days for at least 12 weeks; 3) report having pain rated at least 20 mm on a 100‐mm visual analog scale; 4) have <64° of dorsiflexion range of motion (ROM) of the first MTP joint 19; 5) have pain upon palpation of the dorsal aspect of the first MTP joint; 6) be able to walk household distances (>50 meters) without the aid of a walker, crutches, or cane; 7) be willing to attend the Health Sciences Clinic at La Trobe University (Melbourne, Victoria) on 2 occasions and have their foot radiographed; 8) be willing to not receive additional interventions (such as physical therapy, foot orthoses, shoe modifications, intraarticular injections, or surgery) for first MTP joint pain during the course of the study; and 9) be willing to discontinue taking all medications to relieve pain at their first MTP joint (analgesics and nonsteroidal antiinflammatory medications, except paracetamol up to 4 gm/day) for at least 14 days prior to the baseline assessment and during the study period.

Exclusion criteria for participants in this study were 1) pregnancy; 2) previous surgery on the first MTP joint; 3) significant deformity of the first MTP joint including hallux valgus (grade of 3 or 4, using the Manchester Scale) 20, 21; 4) presence of 1 or more conditions within the foot or ankle that could confound pain and functional assessments of the first MTP joint, such as metatarsalgia, plantar fasciitis, predislocation syndrome, Achilles tendinopathy, and degenerative joint disease (other than the first MTP joint); 5) presence of any systemic inflammatory condition, such as inflammatory arthritis, rheumatoid arthritis, ankylosing spondylitis, psoriatic arthritis, reactive arthritis, septic arthritis, acute pseudogout, gout, or any other connective tissue disease; 6) any medical condition that, in the opinion of the investigators, made the participant unsuitable for inclusion (e.g., severe progressive chronic disease, malignancy, clinically important pain in a part of the musculoskeletal system other than the first MTP joint, or fibromyalgia); 7) cognitive impairment (defined as a score of <7 on the Short Portable Mental Status Questionnaire) 22; 8) intraarticular injections into the first MTP joint in the previous 6 months; 9) currently wearing contoured foot orthoses (although flat insoles were permitted); 10) currently wearing specialized footwear (footwear that has been custom‐made or “prescribed” by a health care practitioner); 11) currently wearing shoes that would not be able to accommodate a foot orthosis; or 12) older adults with a history of recurrent falls (defined as 2 or more falls in the previous 12 months), as there is some evidence that rocker‐sole shoes may have short‐term detrimental effects on balance 23.

Participants were recruited by 1) radio advertisements; 2) advertisements placed in local newspapers, magazines, and social media; 3) posters placed at health care facilities, gymnasiums, senior citizens’ centers, fun runs, and markets; and 4) mail‐out advertisements to patients attending the La Trobe University Health Sciences clinic and to local podiatry clinics. Baseline testing was performed between February and October 2014.

#### Clinical and radiographic assessment

At baseline, participants underwent a clinical assessment, including measurements of height, weight, and body mass index; foot posture (using the Foot Posture Index [FPI]) 24; passive, non*–*weight‐bearing dorsiflexion ROM at the first MTP joint using a flexible, plastic hand‐held goniometer 25; and observation to determine the presence or absence of pain on palpation, a dorsal exostosis, joint effusion, pain during motion, a hard‐end feel when the joint was fully dorsiflexed, and crepitus during movement. The reliability of these assessments has previously been documented 19. Footwear was assessed using the Footwear Assessment Form 26.

The presence or absence of radiographic first MTP joint OA was determined using a radiographic atlas developed by Menz et al 27. The atlas incorporates weight‐bearing dorsoplantar and lateral radiographs to document the presence of OA based on observations of osteophytes and joint space narrowing. Osteophytes were recorded as absent (score 0), small (score 1), moderate (score 2), or severe (score 3). Joint space narrowing was recorded as none (score 0), definite (score 1), severe (score 2), or joint fusion (score 3). The atlas has been shown to have good to excellent intra‐ and interrater reliability for grading first MTP joint OA (κ = 0.64–0.95) 27.

#### Interventions

**Figure 1:**
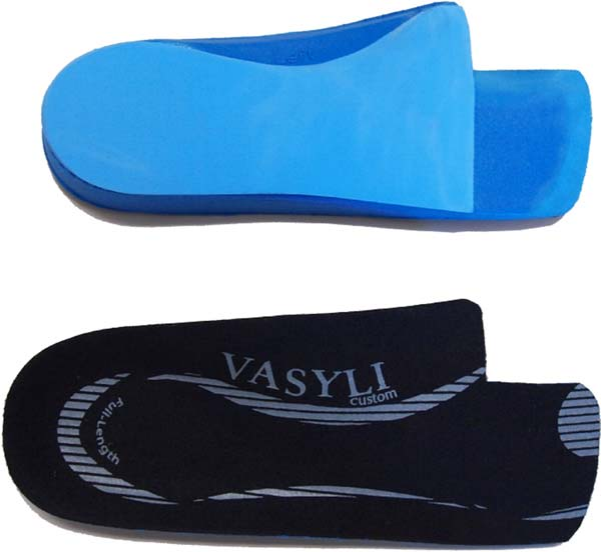
Prefabricated foot orthoses used in the trial. **Top,** plantar surface of left foot orthosis; **bottom,** dorsal surface of right foot orthosis. Reproduced from ref. 18. Color figure can be viewed in the online issue, which is available at http://onlinelibrary.wiley.com/journal/doi/10.1002/acr.22743/abstract.

The prefabricated foot orthoses group received a pair of foot orthoses (Vasyli Custom Medium Density) that were modified using a similar approach to that described by Welsh et al 8. All orthoses were full length, but were modified by adding a cut‐out section beneath the first metatarsal and trimming the distal edge to the level of the second to fifth toe sulci (Figure 1). In participants with pronated feet (defined as an FPI score of >7) 28, full‐length 4‐degree medial (varus) wedges were applied to the underside of the foot orthoses until there was a reduction in the FPI score of at least 2 points 8. The wedge was gradually bevelled so that it extended to the proximal margin of the cut‐out section beneath the first metatarsal. This occurred for 2 participants.

**Figure 2:**
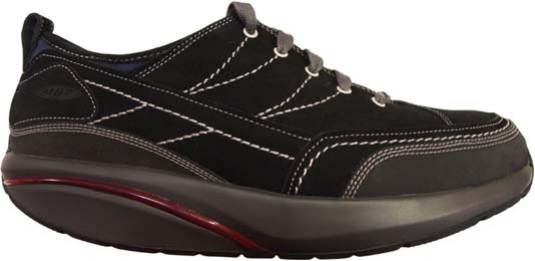
Matwa model footwear (Masai Barefoot Technology). Reproduced from ref. 18. Color figure can be viewed in the online issue, which is available at http://onlinelibrary.wiley.com/journal/doi/10.1002/acr.22743/abstract.

The rocker‐sole footwear group was provided with a pair of appropriately sized rocker‐sole shoes (MBT, Matwa model). This shoe is characterized by a rounded sole in the anteroposterior direction and a soft cushioned heel (Figure 2). Across the full size range, the radius of curvature of the MBT shoe is on average 33 cm overall, 18 cm at the forefoot, 43 cm at the midfoot, and 11 cm at the heel 29. After commencing the study, the MBT shoe we used (the “Mahuta” model) was discontinued by the company and replaced with the “Matwa” model, resulting in 4 participants receiving the Mahuta and 42 receiving the Matwa. However, both models had the same sole curvature and only differed slightly in relation to the aesthetics of the upper.

#### Gait analysis

Both groups underwent the same biomechanical assessment. However, for the prefabricated foot orthoses group, comparisons were made when wearing their own shoes (with and without the prefabricated orthoses), while for the rocker‐sole shoe group, comparisons were made between their own shoes and the rocker‐sole shoes. The order of testing was randomized. After a familiarization period of walking 250 meters, participants completed 4 walking trials for each footwear condition over an 8‐meter distance. To exclude the effect of acceleration and deceleration steps, only the middle 4 steps from each trial were included for analysis. An average recording was determined from 16 steps for each condition. Walking speed was intentionally not controlled for, in order to provide insights into how participants would function under real‐world conditions.

**Figure 3:**
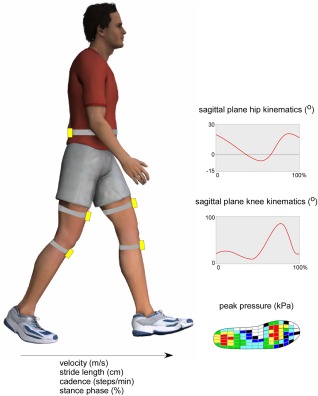
Gait analysis system. Color figure can be viewed in the online issue, which is available at http://onlinelibrary.wiley.com/journal/doi/10.1002/acr.22743/abstract.

Spatiotemporal parameters and sagittal plane peak‐to‐trough ROM of the hip and knee joints during gait were recorded using a wireless, wearable sensor motion analysis system (LEGSys, Biosensics). This system consists of accelerometers and gyroscopes attached with Velcro straps to each lower leg and thigh (Figure 3). The method for calculation of the spatiotemporal parameters of gait is described in detail elsewhere 30. To summarize, the gait phases are determined from the precise events of heel‐strike (initial foot contact) until toe‐off (terminal foot contact). These events are extracted from gyroscopes attached to each shank through a local minimal peak detection scheme. Based on each participant's height and using a biomechanical model, spatial parameters (i.e., stride length and stride velocity) and kinematics (hip and knee) are estimated by integration of the angular rate of rotation of the thigh and shank relative to the waist sensor. Gait analysis with this system has been validated in healthy young controls 30 and older people 31 and has been shown to exhibit acceptable reliability 32.

Peak plantar pressure under the hallux, lesser toes, first MTP joint, second to fifth MTP joints, midfoot, and heel were measured with the in‐shoe Pedar system (Novel GmbH), a reliable, valid, and accurate measure of in‐shoe pressure 33, 34, 35. The Pedar insoles are approximately 2‐mm thick and consist of 99 capacitive pressure sensors arranged in grid alignment. Plantar pressure data were sampled at a frequency of 50 Hz.

#### Statistical analysis

Statistical analysis was undertaken using SPSS, version 22.0. The most symptomatic foot was selected as the index foot for all analyses, or in the case of equivalent symptoms in both feet, the right foot was selected. All data were explored for normality using the skewness statistic (−1 to 1). To evaluate the effects of the interventions (i.e., prefabricated orthoses and rocker‐sole shoes) compared to participants’ own footwear, a series of within‐group, paired *t*‐tests were conducted. To compare the effects of prefabricated orthoses and rocker‐sole shoes, between‐group analyses of covariance were conducted with the intervention group and participants’ own footwear scores entered as independent variables 36. The effect size for within‐group comparisons was calculated using Cohen's *d*, and the following interpretation of effect size was used: negligible (<0.15), small (≥0.15 to <0.40), medium (≥0.40 to <0.75), large (≥0.75 to <1.10), and very large (≥1.10) 37. Adjusted mean differences and 95% confidence intervals were calculated for between‐group comparisons.

## RESULTS

#### Participants

A total of 102 participants were randomized into the study. Five withdrew prior to the baseline assessment, leaving 97 who underwent gait analysis. Characteristics of these participants are reported in Table 1. Due to technical issues with data collection, spatiotemporal/kinematic data were missing from 9 participants, and plantar pressure data were missing from 7 participants. Furthermore, upon initial screening of the data, it was noted that there were 3 significant outliers for peak pressure under the first MTP joint, where extremely high peak pressures were registered for 1 or 2 individual sensors on the most medial edge of the insole. Because the pressure readings obtained from these sensors were unilateral and markedly higher than adjacent sensors, it was concluded that they were due to the Pedar insole being folded or compressed against the medial upper of the shoe. For this reason, first MTP joint peak pressure data from these 3 participants were excluded from the analysis. Therefore, complete spatiotemporal/kinematic and plantar pressure data were available from 88 and 87 participants, respectively.

**Table 1:** Participant characteristics^a^

	Orthoses group (n = 51)	Rocker‐sole group (n = 46)
Demographics and anthropometrics		
Age, years	57.0 ± 11.2	56.5 ± 11.1
Female, no. (%)	28 (54.9)	28 (60.9)
Height, cm	166.2 ± 8.8	166.3 ± 8.3
Weight, kg	80.7 ± 15.0	78.5 ± 13.3
Body mass index, kg/m^2^	29.2 ± 4.8	28.4 ± 4.5
Clinical features		
Pain duration, median (range) months	36 (4–360)	30 (6–420)
Foot Posture Index, mean ± SD (range)	3.0 ± 2.5 (−2 to 11)	3.4 ± 2.2 (−2 to 10)
First MTP joint ROM, degrees	39.8 ± 12.7	40.5 ± 13.0
Pain on palpation, no. (%)	51 (100.0)	46 (100)
Palpable dorsal exostosis, no. (%)	49 (96.1)	45 (97.8)
Joint effusion, no. (%)	17 (34.0)	16 (34.8)
Pain on motion of first MTP joint, no. (%)	49 (96.1)	41 (91.1)
Hard‐end feel when dorsiflexed, no. (%)	46 (90.2)	39 (84.8)
Crepitus, no. (%)	35 (68.6)	30 (65.2)
Radiographic features, no. (%)^b^		
Dorsal osteophytes	48 (96.0)	38 (84.0)
Dorsal joint space narrowing	43 (86.0)	36 (80.0)
Lateral osteophytes	43 (86.0)	35 (77.8)
Lateral joint space narrowing	42 (84.0)	38 (84.4)
Radiographic first MTP joint OA^c^	38 (79.2)	30 (66.7)
Footwear characteristics		
Walking/athletic/Oxford shoe, no. (%)	32 (62.7)	26 (56.5)
Mary Jane/court shoe/boot, no. (%)	9 (17.6)	13 (28.2)
Sandal/slipper/moccasin, no. (%)	10 (19.6)	7 (15.2)
Heel height, mm	23.8 ± 7.9	22.4 ± 10.5
Forefoot height, mm	12.2 ± 5.1	12.2 ± 5.7
Sole flexion point, no. (%)		
At level of MTP joints	35 (68.6)	28 (60.9)
Proximal to MTP joints	10 (19.6)	12 (26.1)
Distal to MTP joints	6 (11.8)	6 (13.0)

a Values are the mean ± SD unless indicated otherwise. MTP = metatarsophalangeal; ROM = range of motion; OA = osteoarthritis.

b Score >0 using atlas in ref. 27.

c At least one score of 2 for osteophytes or joint space narrowing from either view, using case definition from atlas in ref. 27.

#### Spatiotemporal and kinematic data

Spatiotemporal and kinematic data are shown in Table 2. Compared to participants’ own footwear, the orthoses had minimal effects on spatiotemporal or kinematic parameters, with a reduction in velocity (Cohen's *d* = 0.14; negligible effect) and knee ROM (*d* = 0.36; small effect) observed, while the rocker‐sole shoes resulted in reduced cadence (*d* = 0.26; small effect), percentage of the gait cycle spent in stance phase (*d* = 0.44; medium effect), and reduced sagittal plane hip ROM (*d* = 0.44; medium effect). Between‐group comparisons indicated that the percentage of the gait cycle spent in stance phase and sagittal plane hip ROM was lower in the rocker‐sole shoe group compared to the orthoses group.

**Table 2:** Effects of orthoses and rocker‐sole shoes on spatiotemporal/kinematic and plantar pressure parameters^a^

	Orthoses group^b^			Rocker‐sole group^b^			Between‐group comparisons	
	Own footwear	Own footwear +orthoses	*P*	Own footwear	Rocker‐sole footwear	*P*	Adjusted mean difference (95% CI)^c^	*P*
Spatiotemporal/kinematic								
Velocity (minutes/second)	1.06 ± 0.15	1.04 ± 0.14	0.039	1.04 ± 0.13	1.00 ± 0.14	0.075	−0.02 (−0.06, 0.02)	0.374
Stride length (meters)	1.16 ± 0.15	1.15 ± 0.14	0.280	1.11 ± 0.13	1.10 ± 0.14	0.408	−0.02 (−0.06, 0.02)	0.396
Cadence (steps/minute)	109.72 ± 8.09	108.46 ± 8.42	0.055	112.02 ± 8.65	109.85 ± 8.54	0.015	−0.53 (−2.58, 1.53)	0.610
Stance phase (%)	59.69 ± 2.03	59.90 ± 2.14	0.479	59.66 ± 1.77	58.84 ± 2.03	0.021	−1.04 (−1.84, −0.25)	0.010
Sagittal knee ROM	60.89 ± 8.30	57.94 ± 8.31	0.027	59.75 ± 11.69	59.06 ± 9.71	0.576	1.76 (−1.27, 4.78)	0.252
Sagittal hip ROM	47.93 ± 5.03	47.61 ± 5.95	0.587	46.44 ± 4.90	44.46 ± 4.26	0.006	−2.11 (−3.80, −0.42)	0.015
Plantar pressure (kPa)								
Hallux	231.22 ± 92.94	243.11 ± 98.95	0.120	244.11 ± 92.47	252.68 ± 112.14	0.533	−1.42 (−31.49, 28.65)	0.925
Lesser toes	116.94 ± 39.40	139.50 ± 37.51	< 0.001	131.90 ± 56.04	126.25 ± 37.92	0.502	−19.21 (−33.31, −5.10)	0.008
First MTP joint	161.99 ± 54.44	132.95 ± 51.98	< 0.001	166.06 ± 50.44	146.19 ± 39.48	0.002	10.56 (−3.07, 24.20)	0.127
Second through fifth MTP joints	223.67 ± 57.15	236.89 ± 63.26	0.056	223.45 ± 60.83	173.69 ± 48.26	< 0.001	−63.08 (−82.96, −43.20)	< 0.001
Midfoot	95.89 ± 32.51	109.61 ± 29.13	< 0.001	86.43 ± 29.36	90.54 ± 23.78	0.356	−14.21 (−23.49, −4.92)	0.003
Heel	226.78 ± 69.71	187.67 ± 34.42	< 0.001	213.51 ± 49.05	174.11 ± 37.36	< 0.001	−10.01 (−23.38, 3.81)	0.153

a Values are the mean ± SD. 95% CI = 95% confidence interval; ROM = range of motion; MTP = metatarsophalangeal.

b Within‐group comparisons.

c Mean difference between interventions, adjusted for own footwear (control) condition.

#### Plantar pressure data

**Figure 4:**
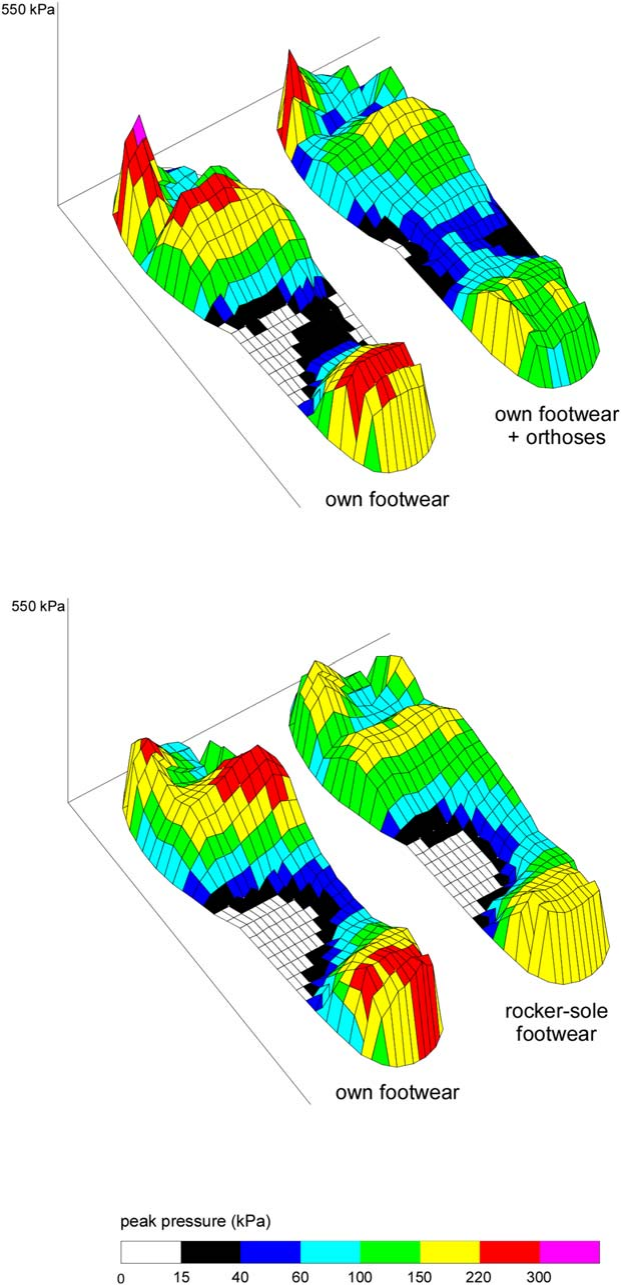
Typical plantar pressure recordings taken from a participant allocated to the orthoses group (**top**) and to the rocker‐sole footwear group (**bottom**). Images represent the mean of 8 steps for the index (**right**) foot. Note the reduced peak pressure under the forefoot and heel, and increased pressure under the midfoot associated with both interventions compared to the participant's own footwear.

Typical examples of peak pressure recordings are shown in Figure 4, and complete peak plantar pressure data are shown in Table 2. Compared to participants’ own footwear, the orthoses increased peak pressure under the lesser toes (*d* = 0.59; medium effect) and midfoot (*d* = 0.45; medium effect), and decreased peak pressure under the first MTP joint (*d* = 0.55; medium effect) and heel (*d* = 0.72; medium effect), while the rocker‐sole shoes decreased peak pressure under the first MTP joint (*d* = 0.44; medium effect), second to fifth MTP joints (*d* = 0.92; large effect), and heel (*d* = 0.91; large effect). Between‐group comparisons indicated that the peak pressure under the lesser toes, second to fifth MTP joints, and midfoot was lower in the rocker‐sole shoes compared to the orthoses, but there was no difference in peak pressure under the first MTP joint.

## DISCUSSION

The objective of this study was to compare the immediate biomechanical effects of prefabricated foot orthoses and rocker‐sole footwear in people with first MTP joint OA. Our findings indicate that both interventions were effective at reducing peak pressure beneath the first MTP joint, which may be one of the mechanisms responsible for their apparent beneficial effects in the treatment of OA affecting this joint. However, they appear to achieve this through different mechanisms. The prefabricated orthoses had minimal effect on spatiotemporal or kinematic parameters, while the rocker‐sole footwear resulted in reduced cadence, percentage of the gait cycle spent in stance phase, and sagittal plane hip ROM. Plantar pressure assessment also revealed that the prefabricated foot orthoses produced an increase in peak pressure under the lesser toes and midfoot and a decrease under the heel, while the rocker‐sole shoes were associated with decreased peak pressure under the second to fifth MTP joints and heel.

The prefabricated orthoses in this study were modified by the addition of a cut‐out section beneath the first metatarsal, as described by Welsh et al 8. The rationale behind this approach is to facilitate first ray plantarflexion, thereby allowing the proximal phalanx to dorsiflex on the first metatarsal head and minimize joint compression during propulsion 6. However, the gait analysis component of the study by Welsh et al found no differences in first MTP joint dorsiflexion when orthoses were worn, despite participants reporting a reduction in symptoms. Our in‐shoe plantar pressure data suggest that orthoses may instead achieve their apparent beneficial effects by redistributing load away from the first MTP joint, possibly by shifting it toward the medial longitudinal arch during midstance and toward the lesser toes during propulsion. Increased midfoot load appears to be a consistent and predictable effect of wearing orthoses, which contour the arch 38, 39. However, the shift in load toward the lateral toes observed in this study is a novel finding and may be specific to the style of orthosis we used (incorporating a cut‐out section beneath the first metatarsal) and/or the condition being studied (first MTP joint OA).

The biomechanical effects of rocker‐sole footwear have been examined in several studies, but none have specifically examined individuals with first MTP joint OA. Our observation of reduced hip joint ROM is consistent with previous investigations using a variety of rocker‐sole designs 13, 40, 41, 42, and has primarily been attributed to the adoption of a shorter stride length. In our study, stride length was not significantly altered when wearing the rocker‐sole shoes. However, there was a reduction in cadence and a trend (*P* = 0.08) toward reduced velocity, both of which may reflect the adoption of a “cautious” gait pattern that has been shown to result in reduced sagittal plane hip motion during gait 43. The combination of a posterior heel rocker, shock‐absorbing heel, and anterior rocker in the MBT shoe may also have a direct influence on hip motion, as less hip flexion may be required for the foot to clear the ground in preparation for initial heel contact 40, the decrease in vertical ground reaction force during contact phase 42 may reduce the internal hip extensor moment, and the relatively “passive” push‐off may require less hip extension during propulsion.

Consistent with several previous studies of a range of rocker‐sole shoes 44, 45, 46, the in‐shoe plantar pressure evaluation revealed a significant reduction in forefoot peak pressure. This finding, combined with our observation of a smaller relative proportion of the gait cycle spent in stance phase, suggests that this style of footwear facilitates forward momentum by enabling the body's center of mass to passively “roll over” the base of support, rather than achieving propulsion through ankle power generation at push‐off. Indeed, studies of gait kinetics when wearing rocker‐sole footwear have reported reductions in peak internal ankle plantarflexor moment 42 and plantarflexor power generation 41 during late stance phase, which is indicative of reduced concentric function of the triceps surae. Given that a reduction in forefoot pressures has been shown to be associated with pain relief in people with forefoot pain 47, it is possible that such a change may also be therapeutically beneficial in people with symptomatic first MTP joint OA by offloading the painful area and reducing joint compression.

The findings of this study need to be considered in the context of several design limitations. First, it was not possible to blind participants to their intervention allocation. Second, the observed changes are immediate effects only, as all gait assessments were performed at the baseline assessment. Although we allowed participants a familiarization period to adapt to their orthoses and footwear, we acknowledge that the effects of the interventions are likely to change over time. Indeed, Stöggl et al 48 have shown that the gait variability induced by MBT shoes significantly reduces after 10 weeks of daily wear, suggesting that some degree of habituation occurs. Third, our gait analysis technique did not allow for in‐shoe assessment of first MTP joint kinematics, as this requires the permanent modification of the upper of the shoe to enable placement of reflective markers or electromagnetic sensors. This approach can compromise the structural integrity of the shoe 49 and is clearly not feasible in the context of a prospective trial where participants are expected to wear the shoes during daily activities over several weeks. Fourth, the wearable sensor motion analysis system we used is restricted to sagittal plane evaluation of the knee and hip. Finally, an inherent limitation of commercially available in‐shoe plantar pressure measurement systems such as the Pedar is that they only measure force perpendicular to the sensor surface. Therefore, the accuracy of measurements made along curved surfaces (such as the medial arch of foot orthoses) may be limited.

In summary, this study has shown that prefabricated foot orthoses and rocker‐sole footwear are effective at reducing peak pressure under the first MTP joint in people with first MTP joint OA; however, they appear to achieve this through different mechanisms. The planned 12‐week followup will determine whether these interventions are acceptable to participants and are effective at reducing joint pain.

## AUTHOR CONTRIBUTIONS

All authors were involved in drafting the article or revising it critically for important intellectual content, and all authors approved the final version to be submitted for publication. Dr. Menz had full access to all of the data in the study and takes responsibility for the integrity of the data and the accuracy of the data analysis.

**Study conception and design**. Menz, Levinger, Roddy, Munteanu.

**Acquisition of data.** Menz, Auhl, Tan, Munteanu.

**Analysis and interpretation of data.** Menz, Auhl, Tan, Levinger, Roddy, Munteanu.
